# An Il12-Il2-Antibody Fusion Protein Targeting Hodgkin's Lymphoma Cells Potentiates Activation Of Nk And T Cells For An Anti-Tumor Attack

**DOI:** 10.1371/journal.pone.0044482

**Published:** 2012-09-18

**Authors:** Tobias Jahn, Martin Zuther, Björn Friedrichs, Claudia Heuser, Stefan Guhlke, Hinrich Abken, Andreas A. Hombach

**Affiliations:** Department I of Internal Medicine and Center for Molecular Medicine Cologne (CMMC), University of Cologne, Cologne, Germany; University of Cape Town, South Africa

## Abstract

Successful immunotherapy of Hodgkin's disease is so far hampered by the striking unresponsiveness of lymphoma infiltrating immune cells. To mobilize both adoptive and innate immune cells for an anti-tumor attack we fused the pro-inflammatory cytokines IL2 and IL12 to an anti-CD30 scFv antibody in a dual cytokine fusion protein to accumulate both cytokines at the malignant CD30^+^ Hodgkin/Reed-Sternberg cells in the lymphoma lesion. The tumor-targeted IL12-IL2 fusion protein was superior in activating resting T cells to amplify and secrete pro-inflammatory cytokines compared to targeted IL2 or IL12 alone. NK cells were also activated by the dual cytokine protein to secrete IFN-γ and to lyse target cells. The tumor-targeted IL12-IL2, when applied by i.v. injection to immune-competent mice with established antigen-positive tumors, accumulated at the tumor site and induced tumor regression. Data demonstrate that simultaneous targeting of two cytokines in a spatial and temporal simultaneous fashion to pre-defined tissues is feasible by a dual-cytokine antibody fusion protein. In the case of IL12 and IL2, this produced superior anti-tumor efficacy implying the strategy to muster a broader immune cell response in the combat against cancer.

## Introduction

Therapy of classical Hodgkin's lymphoma and other CD30^+^ lymphomas has considerably improved during the last two decades [1]; the long-term toxicity of current regimens, however, is still strikingly high, providing a need for alternative strategies. Targeted immunotherapy seems to be well suited in this situation and CD30 to be a good target since the expression pattern of CD30 is restricted and well-characterized antibodies for targeting are available. Immunotherapy of Hodgkin's lymphoma, however, has to take into account that the malignant CD30^+^ Hodgkin/Reed-Sternberg cells (H/RS) persist in small numbers in the lymphoma lesion and are accompanied by massive infiltrations with benign cells [2]–[5]. H/RS cells secrete a variety of cytokines and chemokines favoring a T helper-2 (Th2) immune response which likely contributes to disease progression through restraining cellular reactivity [3],[6]–[10]. Despite a variety of infiltrating immune cells, the overall immune response in Hodgkin's lymphoma patients resembles an acquired cellular immune deficiency [7] with Th2 polarization and elevated IL10 serum levels, both associated with a poor prognosis [11]. A major aim in the immunotherapy of Hodgkin's lymphoma is therefore to break T cell unresponsiveness, in particular in the vicinity of H/RS cells. Cytokines which reverse the polarized immune response are thought to be good candidates to re-activate an anti-tumor immune response; antibody-targeted cytokines that accumulate in the lymphoma lesion are thought to be more efficacious than un-modified cytokines. This provides the rationale to target cytokines towards H/RS tumor cells by use of antibody-cytokine fusion proteins. Local re-activation of the immune response seems to be beneficial in the therapy of Hodgkin's lymphoma since bi-specific antibody-mediated activation of NK cells along with T cells in the lymphoma lesion showed some therapeutic effect [12]–[14]. A recombinant bi-specific antibody targeting CD30 on Hodgkin's lymphoma cells and the Fc γ receptor (CD64) on monocytes triggers CD64 mediated effector functions [15].

The therapeutic efficacy of antibody-targeted cytokines, however, strongly depends on an optimized molecular design which needs to be evaluated for each cytokine. For instance, each individual domain in the fusion protein affects structural properties, the binding avidity and retention time in the tumor tissue, the pharmacodynamics and pharmacokinetics, each of which are therapeutically relevant properties. The therapeutic situation is even more complex since many tumor cells shed the targeted cell-surface-antigen in substantial amounts which competes in binding with the tumor-cell-bound antigen. This is the case for CD30 in Hodgkin's lymphoma giving rise to substantial serum levels of soluble CD30 (sCD30). Favored binding to solid-phase bound antigen in the presence of high amounts of soluble antigen is therefore required. In addition, a number of cytokines cause high systemic toxicity; those cytokines can only be applied when specifically targeted to tumor tissues, delivered by a targeting antibody that is fused to the cytokine, leaving healthy tissues with sub-toxic cytokine concentrations. On the other hand, the functional properties of the cytokine may be diminished when fused to other protein domains. These and other examples make obvious that the therapeutic efficacy of the tumor-targeted cytokine depends, among others, on the binding avidity and the immune-modulatory capacity of the fusion protein.

We here generated and analyzed a panel of antibody-cytokine fusion proteins to target IL2 and IL12 toward CD30^+^ H/RS lymphoma cells in order to locally activate both an adoptive and innate immune response. Dimerization via a constant IgG domain rendered fusion proteins more robust against high concentrations of soluble CD30 when targeting CD30^+^ H/RS cells. To take advantage of the co-operative action of IL2 and IL12 in activating T and NK cells we generated a dual cytokine-antibody fusion protein which delivers both IL2 and IL12 simultaneously to H/RS cells. The anti-CD30 single chain fragment HRS3-scFv [16] is linked to the N-terminus of single chain p40-p35 IL12 (scIL12) which is fused via the IgG1-hinge-CH2CH3 domain to the N-terminus of IL2. This particular design enabled targeting of cooperatively active cytokines to CD30^+^ cells in a spatial and temporal concomitant fashion. The dual cytokine-antibody fusion protein showed superior in activating resting T and NK cells compared to the corresponding fusion proteins containing either IL2 or IL12 and was highly active in a syngenic mouse model. Data demonstrate that simultaneous targeting of co-operating cytokines will be a valuable strategy to muster a broad immune response in the specific immunotherapy of cancer.

## Results

### Anti-Cd30-Antibody-Cytokine Fusion Proteins With Combined Il12-Il2 Cytokine Domains

**Figure 1:**
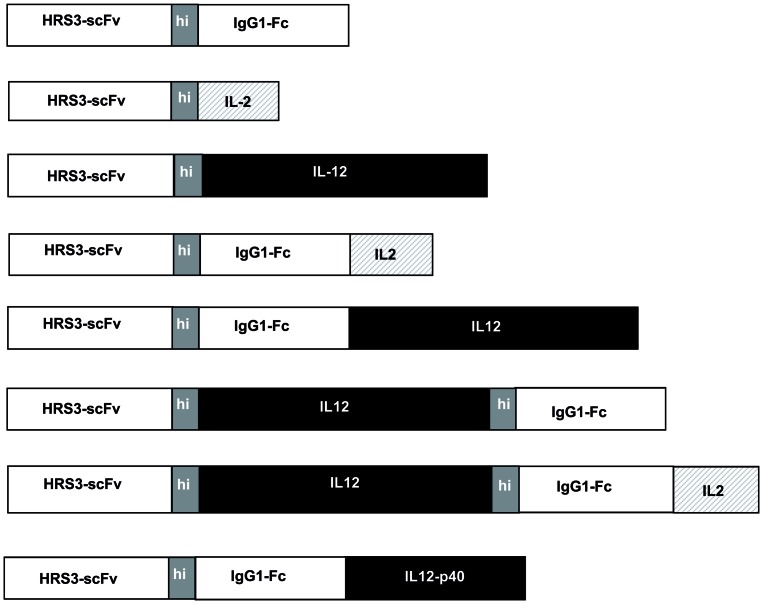
Figure 1. Schematic diagram of the anti-CD30 antibody-cytokine fusion proteins used in the study. IL12 represents the p35–p40 single-chain IL12. hi: IgG1 hinge region; IgG1-Fc: IgG1 CH2CH3.

We generated a series of recombinant anti-CD30 antibody-cytokine fusion proteins, each with the same anti-CD30 HRS3 single chain fragment of variable regions (scFv) antibody for targeting CD30^+^ Hogdkin's lymphoma cells, but with different cytokine domains (Fig. 1). The scFv antibody was linked via the human IgG1 hinge (hi) domain to the IgG1 CH2CH3 (Fc) constant domain or to the IL2 or IL12 cytokine. Alternatively, the cytokine is fused to the C-terminal of the IgG1 Fc domain. To target IL2 and IL12 simultaneously to CD30^+^ tumor cells, both cytokines were linked together in one polypeptide chain and fused to the anti-CD30 antibody for targeting. In particular, the HRS3 scFv antibody was linked via the human IgG1 hinge domain to the murine p35–p40 single chain IL12 which was furthermore linked via the IgG1 hinge-CH2CH3 (Fc) domain to human IL2. The fusion proteins were expressed in 293T cells and secreted into the culture medium. The concentration of IgG Fc-fusion proteins in the culture supernatant was determined by ELISA detection of the common IgG Fc domain and adjusted to equimolar titers.

**Figure 2:**
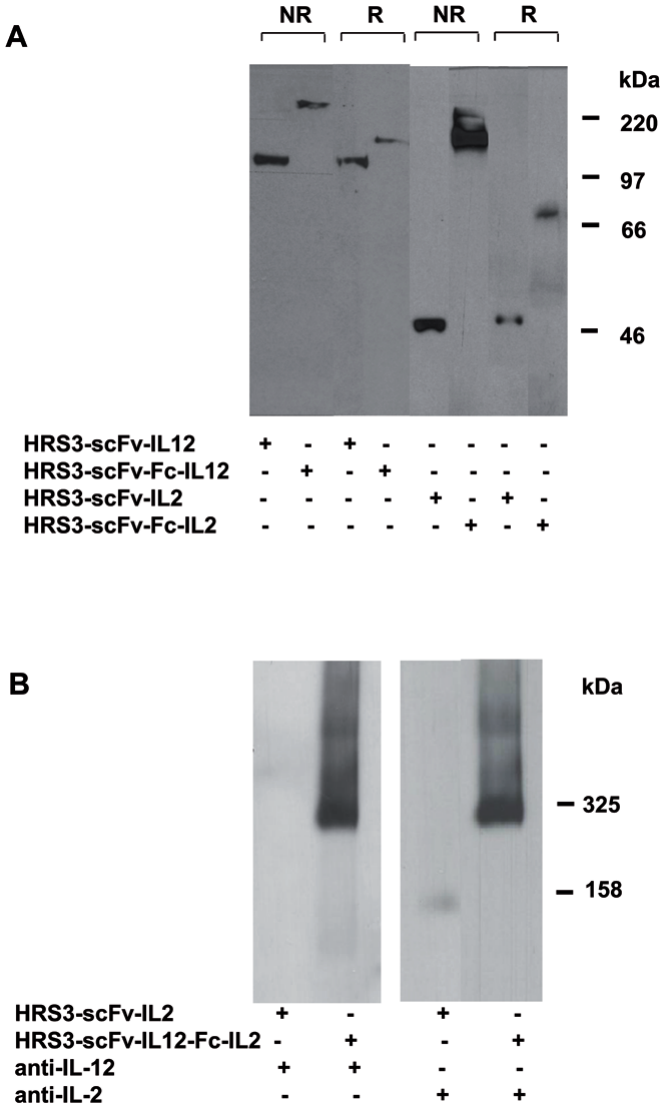
Figure 2. Western blot analysis of the anti-CD30 antibody-cytokine fusion proteins. (A) Supernatants containing HRS3-scFv-IL12 and HRS3-scFv-Fc-IL12 or HRS3-scFv-IL2 and HRS3-scFv-Fc-IL2 fusion proteins, respectively, were separated by SDS-PAGE under reducing (R) and non-reducing (NR) conditions. (B) Supernatants of 293T producer cell lines containing HRS3-scFv-Fc-IL2 and dual cytokine HRS3-scFv-IL12-Fc-IL2 fusion proteins, respectively, were separated by blue native polyacrylamide gel electrophoresis (BN-PAGE). Separated proteins were transferred to PVDF membranes and detected by staining the blots with anti-IL12 and anti-IL2 antibodies, respectively.

Western blot analyses of HRS3-scFv-IL12 and HRS3-scFv-Fc-IL12 fusion proteins under non-reducing conditions revealed proteins of about 110 kDa and 270 kDa; the molecular weight corresponds to the monomeric form of the HRS3-scFv-IL12 and the dimeric form of the HRS3-scFv-Fc-IL12 fusion protein (Fig. 2A). The conclusion was confirmed by analyses under reducing conditions revealing the same 110 kDa band for the HRS3-scFv-IL12 protein and a 135 kDa band corresponding to the monomeric HRS3-scFv-Fc-IL12 protein (Fig. 2A). We obtained similar results for the IL2 containing fusion proteins with about 45 kDa for HRS3-scFv-IL2 and about 75 kDa and 150 kDa for HRS3-scFv-Fc-IL2 under reducing and non-reducing conditions, respectively (Fig. 2A). The observations imply that the IgG1-hinge-Fc domain which provides cysteine-disulfide bonds mediates dimerization of the antibody-cytokine fusion proteins independently of the fused cytokine domain whereas the IgG1 hinge region without Fc constant domain does not. Since polymers of the fusion proteins may impact antibody-mediated binding we analyzed the HRS3-scFv-Fc-IL2 and HRS3-scFv-IL12-Fc-IL2 fusion proteins by native polyacrylamide gel electrophoresis (BN-PAGE). Both proteins were predominantly detected as dimeric molecules with the expected molecular size of about 150 kDa and 290 kDa, respectively (Fig. 2B). Protein detection was specific since the HRS3-scFv-IL12-Fc-IL2 dual cytokine protein was detected by both the anti-IL2 and the anti-IL12 antibody, indicating the presence of both cytokine domains, whereas the HRS3-scFv-Fc-IL2 protein was detected by the anti-IL2 antibody only.

### Antibody-Il12 Fusion Proteins Showed Specific Antibody-Mediated Binding And Targeting *In Vivo*

**Figure 3:**
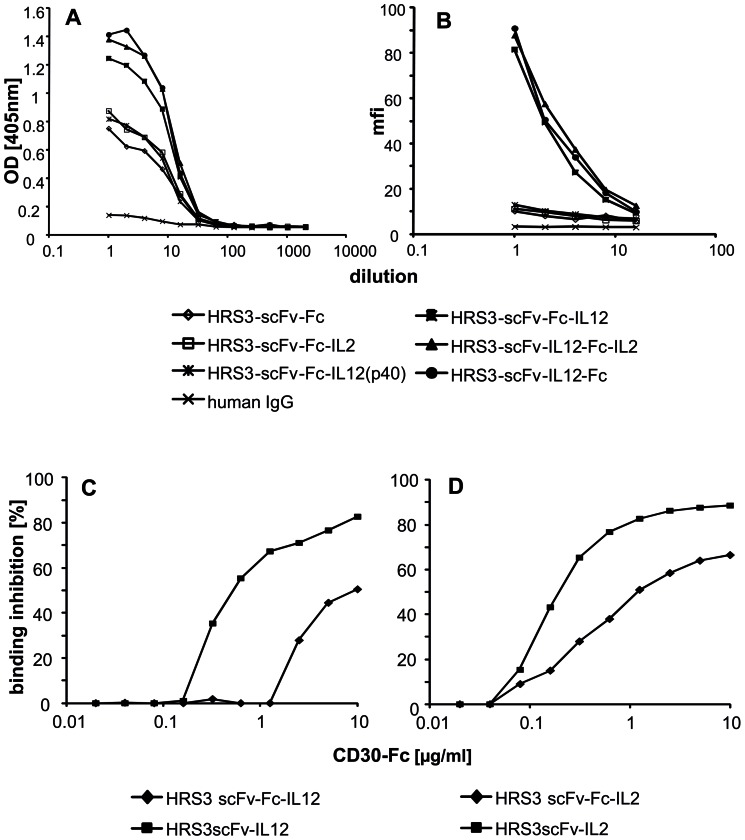
Figure 3. Antigen-specific binding of antibody targeted IL-2 and IL12 fusion proteins. (A) Equimolar amounts of the scFv-Fc fusion proteins were incubated in serial dilutions in micro-titer plates coated with the anti-idiotypic mAb 9G10 which binds to the HRS3 scFv antibody targeting domain of the fusion proteins. For control, same amounts of human IgG1 were added. Bound fusion proteins were detected by an anti-IgG1 antibody directed towards the Fc spacer domain. A representative experiment out of three is shown. (B) CD30^+^ L540cy Hodgkin's lymphoma cells were incubated with serial dilutions of the anti-CD30-Fc fusion proteins or of IgG1 for control. Bound proteins were detected by a PE-conjugated F(ab)_2_ goat anti-human IgG1 antibody. Cells were analyzed by flow cytometry and the mean fluorescence intensity (MFI) was determined. A representative experiment out of three is shown. (C) Equimolar amounts of purified HRS3-scFv-IL12 and HRS3-scFv-Fc-IL12 fusion proteins, respectively, or (D) of HRS3-scFv-IL2 and HRS3-scFv-Fc-IL2 fusion proteins, respectively, were incubated in CD30 coated microtiter plates in the presence of increasing amounts of soluble CD30. Bound proteins were detected by biotinylated anti-IL12 (C) or anti-IL2 (D) antibodies and streptavidin-conjugated POD. Binding inhibition was determined as described in Materials and Methods. An experiment out of two with similar results is shown.

To record specific binding of the Fc-fusion proteins we incubated equimolar amounts in micro-titer plates that were coated with the HRS3 scFv-specific anti-idiotypic mAb 9G10 carrying the internal image of the HRS3 scFv. Bound fusion proteins were recorded by detecting the common human IgG domain of the fusion proteins. Human IgG without HRS3 binding domain served as control. All fusion proteins with the HRS3-scFv domain bound to the anti-idiotypic mAb 9G10 (Fig. 3A). Binding was specific since no binding to an immobilized isotype control mAb was observed (data not shown). We obtained essentially the same results upon binding of the fusion proteins to CD30 expressed on the surface of L540cy Hodgkin's lymphoma cells as recorded by flow cytometry (Fig. 3B).

Antigen shedding by tumor cells results in high concentrations of the soluble target antigen in serum with the potential to neutralize antibody mediated binding thereby limiting antibody targeted therapy of cancer. We addressed how various formats of anti-CD30 cytokine fusion proteins are affected by soluble CD30 (sCD30) in HRS3 scFv antibody mediated binding to solid phase bound CD30. Monomeric scFv and dimeric scFv-Fc fusion proteins were purified as described in Materials and Methods and equimolar amounts were incubated with immobilized CD30 in the presence of serial dilutions of soluble CD30; the binding inhibition was determined and results are summarized in Fig. 3 C, D. There were substantial differences in the ability of the fusion proteins to bind to immobilized CD30 in the presence of competing sCD30. The amount of sCD30 that was required to block binding of the HRS3-scFv-Fc-IL12 fusion protein to solid phase CD30 was at least tenfold higher than those to block binding of the monomeric HRS3-scFc-IL12 (Fig. 3C). Similarly, the HRS3-scFv-Fc-IL2 fusion protein required about fivefold more sCD30 for inhibition than the monomeric HRS3-scFv-IL2 (Fig. 3D). This demonstrates that the dimeric format of the antibody-cytokine fusion protein has substantially higher avidity to solid phase CD30 in presence of soluble CD30.

**Figure 4:**
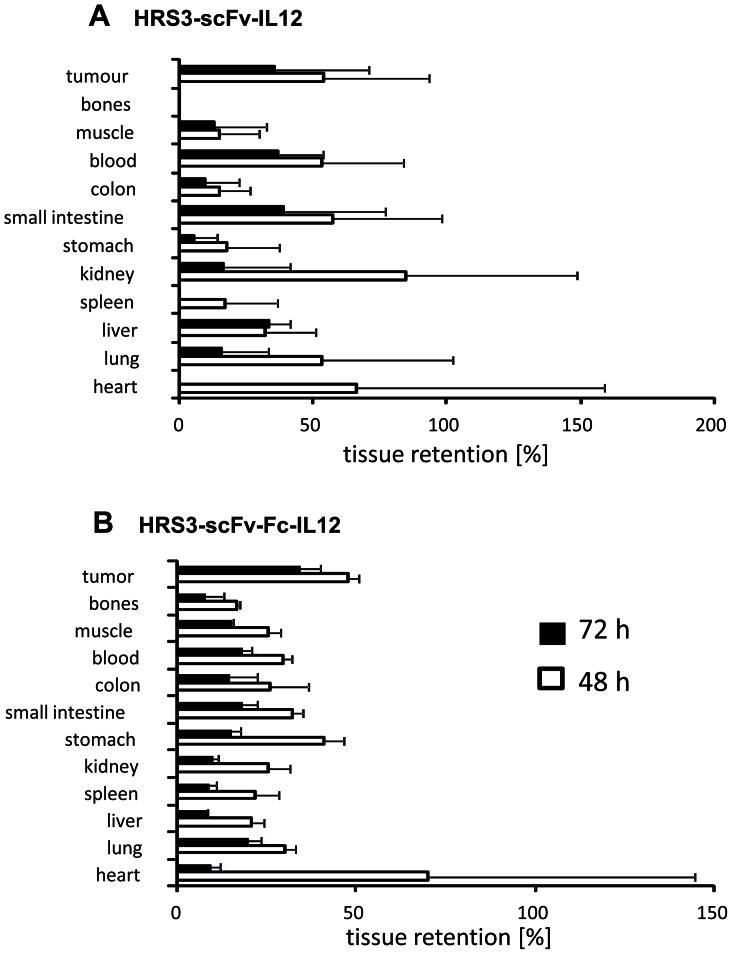
Figure 4. Biodistribution of HRS3-scFv-IL12 and HRS3-scFv-Fc-IL12 fusion proteins. HRS3-scFv-IL12 and HRS3-scFv-Fc-IL12 fusion proteins were purified and labeled with ^131^I as described in Materials and Methods. SCID mice were s.c. transplanted with CD30^+^ L540cy Hodgkin's lymphoma cells (2×10^7^/animal) and animals with established tumors (>0.5 cm^3^) were intravenously injected with ^131^I -labeled fusion proteins. Animals were sacrificed after 24 h, 48 h and 72 h (HRS3-scFv-IL12: 4 mice/group; HRS3-scFv-Fc-IL12: 3 mice/group), respectively, organs were recovered and tissue retention of radioactivity (%) was determined as described in Materials and Methods.

We recorded whether the increased binding avidity of the dimeric HRS3-scFv-Fc-IL12 fusion protein results in improved binding to CD30^+^ tumors in vivo compared to the monomeric HRS3-scFv-IL12 fusion protein. SCID mice were subcutaneously inoculated with CD30^+^ L540 Hodgkin's lymphoma cells giving rise to progressively growing tumors. Radio-labeled fusion proteins were intravenously injected into mice when tumors were established (>0.5 cm3) and the organ distribution of HRS3-scFv-IL12 (Table 1) and HRS3-scFv-Fc-IL12 (Table 2), respectively, was recorded 24 h, 48 h and 72 h after injection. The HRS3-scFv-Fc-IL12 fusion protein was preferentially retained in the tumor tissue, indicated by higher amounts of the initial dose after 72 h, whereas the HRS3-scFv-fusion protein accumulated rather non-specifically in the small intestine, liver and blood (Fig. 4). The biological half-life of the labeled HRS3-scFv-IL12 protein was calculated to be 32 h, those of the HRS3-scFv-Fc-IL12 protein to be 34 h. Data indicate that the monomeric HRS3-scFv-IL12 fusion protein has a higher tissue penetration capacity whereas the dimeric HRS3-scFv-Fc-IL12 fusion protein exhibits improved binding and higher tumor retention. We also determined the serum half life of the HRS3-scFv-IL12-Fc-IL2 dual cytokine fusion protein by injecting three doses of the HRS3-scFv-IL12-Fc-IL2 fusion protein every second day intravenously into mice and use ELISA to record the content of fusion protein in serum samples that were drawn at different time points. Injected IL12-IL2 fusion protein accumulated in the serum reaching its maximum 2 days after the last injection and decreased again. From these data we estimated a biological half-life of about 36 h for the dual cytokine fusion protein, a value similar to those of the fusion proteins containing only an IL12 domain.

**Table 1:** Biodistribution of HRS3-scFv-IL12 fusion protein.

	24 h		48 h		72 h	
organ	mean^1^	SD	mean	SD	mean	SD
heart	0.023	0.041	0.015	0.027	0.000	0.000
lung	0.130	0.086	0.069	0.067	0.020	0.023
liver	0.259	0.288	0.082	0.065	0.086	0.021
spleen	0.509	0.514	0.087	0.053	0.000	0.000
kidney	0.164	0.072	0.139	0.111	0.027	0.040
stomach	0.158	0.128	0.028	0.039	0.009	0.013
sm intestine	0.047	0.024	0.027	0.026	0.018	0.018
colon	0.176	0.252	0.027	0.024	0.017	0.022
blood	0.356	0.159	0.191	0.122	0.132	0.059
muscle	0.028	0.023	0.004	0.005	0.004	0.005
bones	0.052	0.079	0.000	0.000	0.000	0.000
tumor	0.081	0.046	0.044	0.038	0.029	0.028

1 Values represent the mean percentage of injected dose per g tissue (n = 4 mice).

doi:10.1371/journal.pone.0044482.t001

**Table 2:** Biodistribution of HRS3-scFv-Fc-IL12 fusion protein.

	24 h		48 h		72 h	
organ	mean^1^	SD	mean	SD	mean	SD
heart	0.575	0.041	0.404	0.521	0.058	0.026
lung	0.695	0.082	0.212	0.023	0.141	0.039
liver	1.314	0.124	0.278	0.048	0.113	0.013
spleen	1.049	0.301	0.232	0.079	0.097	0.041
kidney	0.723	0.161	0.185	0.056	0.075	0.021
stomach	0.168	0.014	0.069	0.011	0.026	0.008
sm intestine	0.356	0.043	0.114	0.013	0.066	0.023
colon	0.414	0.112	0.108	0.054	0.062	0.045
blood	1.090	0.137	0.323	0.035	0.201	0.052
muscle	0.143	0.028	0.037	0.006	0.023	0.002
bones	0.249	0.047	0.042	0.003	0.021	0.018
tumor	0.266	0.049	0.127	0.01	0.091	0.023

1 Values represent the mean percentage of injected dose per g tissue (n = 3 mice).

doi:10.1371/journal.pone.0044482.t002

### The Il12-Il2 Dual Cytokine-Antibody Fusion Protein Has Superior Power In Activating T And Nk Cells

**Figure 5:**
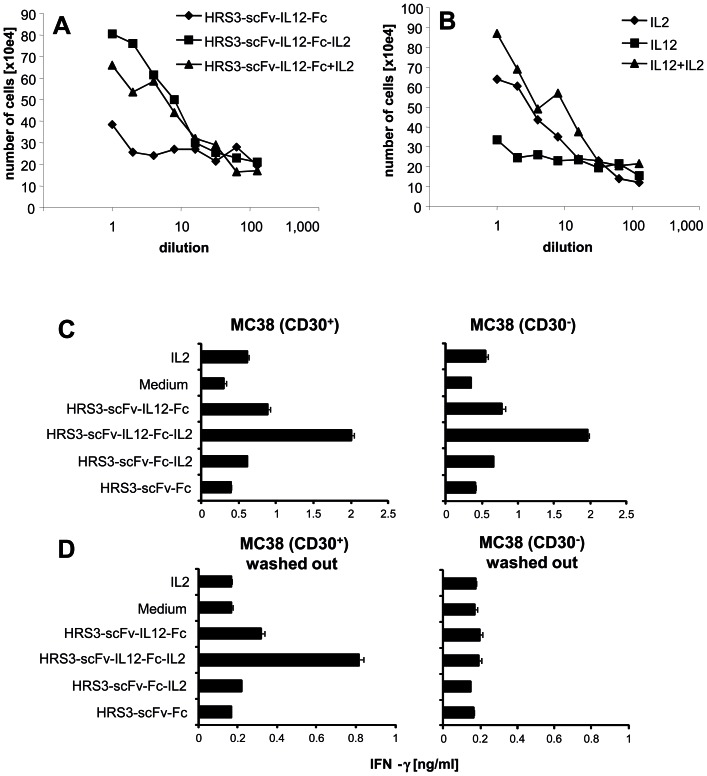
Figure 5. The HRS3-scFv-IL12-Fc-IL2 dual cytokine fusion protein bound to CD30^+^ cells induces T cell amplification and IFN-γ secretion. (A, B) Pre-activated T cells were incubated for 48 hrs with serial dilutions of equimolar amounts of the anti-CD30 cytokine fusion proteins or the cytokines IL2, IL12 or IL2 plus IL12. Viable cells were counted; dead cells were excluded by trypan blue exclusion. (C, D) CD30^+^ and CD30^−^ MC38 cells (5×10^4^ cells/well) were incubated with equimolar amounts of anti-CD30 fusion proteins for 1 h on ice. Unbound fusion proteins were either present during the assay (C) or washed out (D). Pre-activated T cells (5×10^4^ cells) were co-incubated for 48 hrs. IL2 (500 U/ml) was added for comparison. T cell activation was recorded by monitoring IFN-γ secreted into the culture medium. Data represent the mean of triplicates ± standard error of mean (SEM). An experiment out of two with similar results is shown.

Since T and NK cells are crucial mediators of a cellular anti-tumor response, we aimed at activating those cells by the dual cytokine fusion protein in contrast to fusion proteins with one cytokine only. CD3^+^ T cells and CD16^+^ NK cells were isolated from the peripheral blood by negative sorting techniques and incubated with the respective cytokines. As summarized in Fig. 5A, the IL12-IL2 fusion protein induced proliferation of pre-activated T cells whereas the IL12 fusion protein did less. Notably, the combined IL12-IL2 dual cytokine fusion protein enhanced T cell proliferation in a similar fashion as did the combination of IL12 and IL2 fusion proteins. Similar observations were made using the respective cytokines (Fig. 5B). Data show that the antibody targeted, combined IL12-IL2 is highly effective in inducing T cell amplification when added to the cultures.

We next asked whether the dual cytokine protein activates T and NK cells when bound to the membrane of CD30^+^ target cells. CD30^−^ MC38 tumor cells as well as transfected CD30^+^ MC38 cells were incubated with equimolar amounts of the respective fusion proteins. Cells were thoroughly washed to remove unbound proteins and co-incubated with pre-activated T cells; for comparison fusion proteins were added during co-incubation with T cells. As summarized in Fig. 5C, the IL12-IL2 dual cytokine induced T cell activation more effectively when compared to proteins with either IL12 or IL2 domain alone as indicated by the increase in IFN-γ secretion. After extensive washings, the targeted IL12-IL2 dual cytokine still induced IFN-γ secretion in T cells (Fig. 5D). This indicates that the fusion protein has retained its capacity to activate T cells when bound to the surface of CD30^+^ target cells. When incubated with CD30^−^ MC38 cells as control and extensively washed afterwards the IL12-IL2 dual cytokine did not induce IFN-γ secretion of T cells (Fig. 5D). The effect is mediated by the cytokines since the anti-CD30-scFv protein without a cytokine did not induce T cell activation. Taken together, the IL2 and IL12 cytokine domains exhibited synergistic activity in activating T cells when combined in an IL12-IL2 fusion protein and retained their activity when bound via a fused antibody to CD30^+^ target cells.

**Figure 6:**
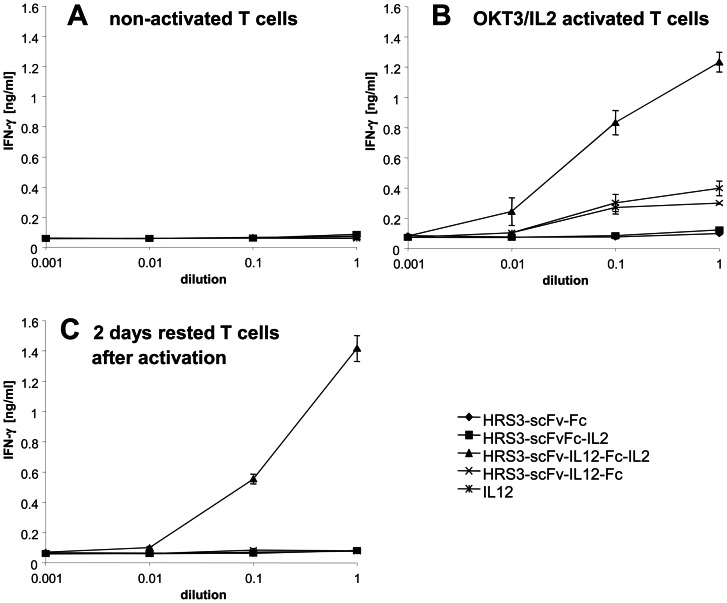
Figure 6. The HRS3-scFv-IL12-Fc-IL2 fusion protein rescues IFN-γ secretion of rested T cells. Non-activated T cells (A), OKT3/IL2 pre-activated T cells (B) and pre-activated T cells that were rested for 2 days without further stimulation (5×10^4^ cells/well each) were incubated with serial dilutions of equimolar amounts of the fusion proteins for 48 hrs. IFN-γ in the culture supernatants was recorded by ELISA. Data represent the mean of triplicates ± standard error of mean (SEM). An experiment out of two with similar results is shown.

**Figure 7:**
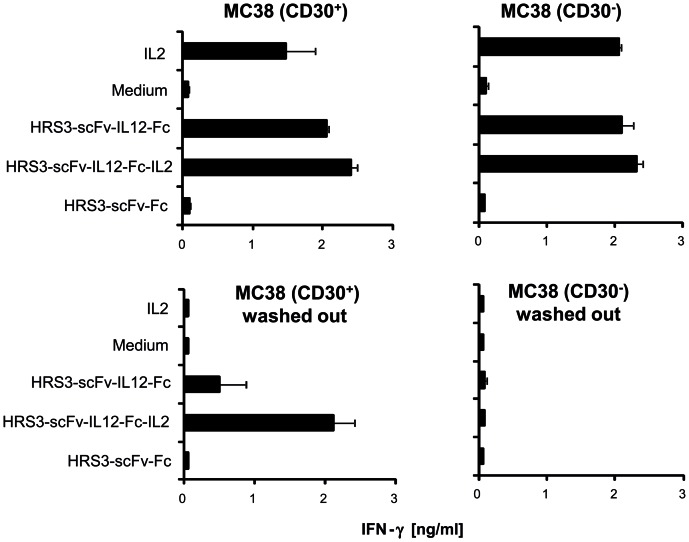
Figure 7. The HRS3-scFv-IL12-Fc-IL2 dual cytokine fusion protein bound to CD30^+^ cells activates resting NK cells to IFN-γ secretion. CD30^+^ and CD30^−^ MC38 cells (5×10^4^ cells/well) were incubated with equimolar amounts of anti-CD30 fusion proteins for 1 h on ice. Unbound fusion proteins were either present during the assay or washed out. Freshly isolated NK cells (5×10^4^ cells) were co-incubated for 48 hrs. IL2 (500 U/ml) was added for comparison. NK cell activation was recorded by monitoring IFN-γ secreted into the culture medium. Data represent the mean of triplicates ± standard error of mean (SEM). An experiment out of two with similar results is shown.

T cells upregulate both IL2 and IL12 receptor expression upon activation which is repressed after removal of the activating stimuli. We therefore asked whether the IL12-IL2 dual cytokine rescues T cell activation after withdrawal from primary stimuli. Resting CD3^+^ T cells, anti-CD3/IL2 activated T cells or T cells which were kept clear from stimuli for 2 days after activation were incubated with the dual cytokine fusion protein and with proteins containing one cytokine only. The dual cytokine rescued IFN-γ secretion of 2 day resting T cells whereas the fusion proteins with IL2 or IL12 only did not (Fig. 6). For comparison, activated T cells increased IFN-γ secretion in the presence of the fusion proteins with IL2, IL12 or both cytokines. T cells secreted most IFN-γ upon incubation with the combined IL12-IL2 cytokine protein. Non-activated T cells were not activated by either fusion protein. Data demonstrate the cooperative activity of the cytokines IL2 and IL12 in the dual cytokine fusion protein in re-activating resting T cells. In addition to T cells, resting NK cells were also activated to secrete IFN-γ upon incubation with the IL12-IL2 dual cytokine (Fig. 7). Equimolar amounts of the IL12 fusion protein induced IFN-γ with similar efficiencies as the IL12-IL2 dual cytokine when added to the target cells. When bound to the surface of CD30^+^ target cells, strikingly, the dual cytokine was much more efficient in activating NK cells.

**Figure 8:**
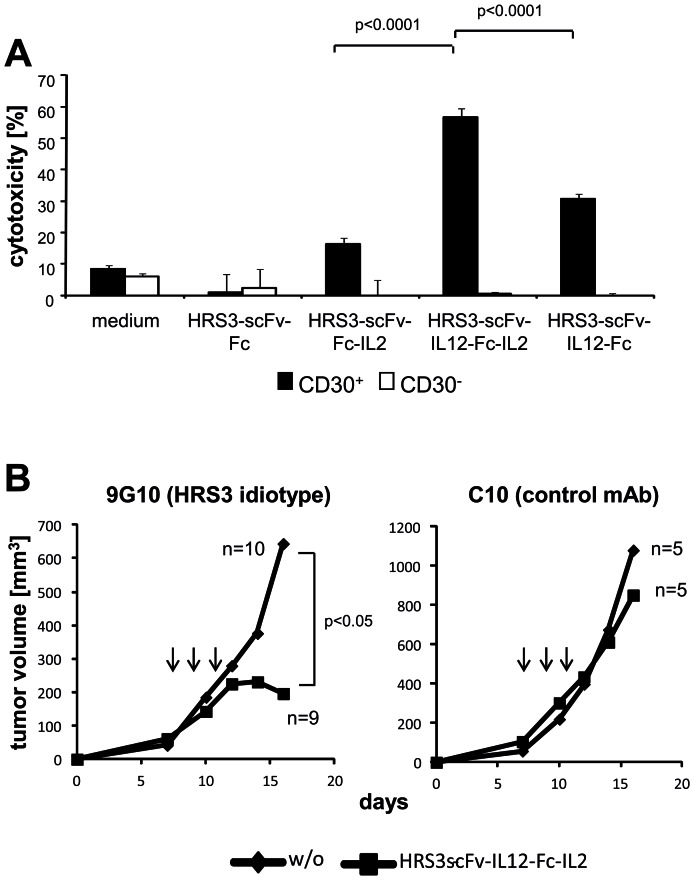
Figure 8. HRS3-scFv-IL12-Fc-IL2 fusion protein improves lysis of CD30^+^ tumor cells and represses tumor growth in immune-competent mice. (A) CD30^+^ and CD30^−^ MC38 tumor cells were pre-incubated for 1 h on ice with equimolar amounts of the indicated fusion proteins. Cells were washed extensively and co-cultured (5×10^4^ cells/well) for 48 hrs in round-bottom micro-titer plates together with freshly isolated, non-activated NK cells (each 5×10^4^ cells/well). Co-incubation with NK cells without fusion protein (medium) served as control. Cytotoxicity against CD30^+^ tumor cells was determined by a XTT-based assay as described in Materials and Methods. Data represent the mean of triplicates ± standard error of mean (SEM). Statistical analyses were performed by Student's T test. A representative experiment out of three is shown. (B) Balb/c mice were transplanted with 9G10 hybridoma cells expressing the HRS3-specific anti-idiotypic mAb on the cell surface (n = 19) or for control with C10 hybridoma cells expressing an IgG1 with irrelevant specificity (n = 10) (2.5×10^6^ cells/mouse). At day 7, when the tumor was fairly established, mice received i. v. injections of the purified HRS3-scFv-IL12-Fc-IL2 fusion protein (50 µg/animal) or the same volume PBS for control (5–10 animals/group). Injections were repeated twice every second day. Tumor volume was recorded every 2–3 days. Mean values are shown; p values were determined by Student's T test.

To record NK cell mediated cytolysis, CD30^+^ and CD30^−^ MC38 target cells were incubated with the fusion proteins, washed and co-incubated with NK cells. Whereas cell-bound IL2 and IL12 fusion proteins induced NK cells to some cytolysis, the cell-bound IL12-IL2 dual cytokine fusion protein induced much higher cytolytic activity toward CD30^+^ MC38 cells (Fig. 8A). Fusion proteins mediated NK cell activation when bound to the surface of CD30^+^ target cells; incubation of target cells without cytokine fusion protein did not induce cytolysis. CD30^−^ cells which did not bind the fusion proteins were not eliminated by NK cells. The proteins itself did not exhibit toxicity as shown by incubation in the absence of NK cells (data not shown).

We asked to explore the anti-tumor activity of the fusion proteins in an immune competent mouse model. To our knowledge there is no adequate model for Hodgkin's lymphoma available; we therefore made use of transplanted syngenic tumors produced by 9G10 hybridoma cells which express the HRS3 specific anti-idiotypic antibody on the cell surface as surrogate antigen. Balb/c mice were subcutaneously injected with syngenic 9G10 cells and for control with hybridoma cells expressing the control mAb C10 of irrelevant specificity. When the tumors were established mice were injected three times intravenously with the purified IL2-IL12 fusion protein and tumor growth was recorded. As summarized in Fig. 8B, the dual cytokine fusion protein substantially suppressed tumor growth compared to mice that received saline solution for control. Inhibition of tumor growth was target-antigen-specific since growth of the C10 hybridoma was not affected by the HRS3-IL12-Fc-IL2 fusion protein.

## Discussion

We previously revealed that IL2 and IL12 act partly overlapping in the activation of T and NK cells [17]; IL2 is a potent inducer of proliferation and IL12 a stimulator of cytokine secretion including IFN-γ. Both cytokines act synergistically with respect to these functions resulting in an improved target cell lysis [17]. To take advantage of the synergistic action, we incorporated IL2 and IL12 into a dual cytokine fusion protein linked to the anti-CD30-scFv antibody for targeting the cytokines toward Hodgkin's lymphoma cells. The two cytokines integrated into the fusion protein synergized in their activities towards T and NK cells which is thought to be of benefit in the targeted immunotherapy of Hodgkin's lymphoma. In particular, the fusion protein induced cytolysis in resting NK cells and re-activated IL2-deprived T cells. These properties were not provided by the corresponding proteins with one cytokine only. In contrast to the dual cytokine protein, simultaneous application of both single-cytokine proteins is likely less effective in delivering both cytokines to the same cell at the same time. Delivery of two synergistically acting cytokines to the same target cell makes the strategy unique compared with other immunotherapeutic approaches applying targeted cytokines or bi-specific antibodies.

The modular composition of the antibody-cytokine fusion proteins facilitates integration of various domains which increase the binding valency by dimerization of the molecule. This is shown by integration of the IgG1 Fc domain which mediates homodimerization of the molecule by cysteine disulfide bonds. Increased valency of the antibody-cytokine fusion protein has consequences for its therapeutic application: a higher avidity, compared to the monomeric form, results (i) in favorable binding to solid phase antigen even in the presence of the soluble antigen, and (ii) lower tissue penetration but higher and specific retention in the targeted tissues *in vivo*. Both consequences were demonstrated by an appropriate experimental setup in this study (cf. Fig. 3C,D; Fig. 4).

Unintended activation of effector cells beyond target sites is a major issue of current immunotherapy regimens. IL2 and IL12 receptors are upregulated by activated T and NK cells and CD30 is transiently expressed on these cells which may result in binding of anti-CD30 fusion proteins and unintended off-target effector cell activation. Whereas dimerized fusion proteins with multiple cytokine domains may have an increased risk of unintended effector cell activation, the antibody binding domains of these proteins bind to tumor cells with higher avidity. Moreover, specific binding is more resistant to blocking by soluble target antigen and specific tissue retention *in vivo* is improved. Taken these properties together, dimerized fusion proteins with a higher valency appear to be more suitable for site specific immunotherapy than the corresponding monomeric proteins. On the other hand CD30 is predominantly expressed on activated Th2 cells [18] that are present in high numbers in the tissue of Hodgkin's lymphoma [19]. Accordingly, targeting Th2 polarized T cells with the anti-CD30-IL12-IL2 dual cytokine fusion protein may additionally modulate the tumor-environment of Hodgkin's lymphoma by shifting T cells to Th1 reactivity.

The simultaneous synergistic action of IL2 and IL12 will re-activate both types of cytolytic effector cells, T and NK cells, in the lymphoma lesion. Increased IFN-γ secretion that is associated with NK and T cell activation, in concert with delivered IL2 and IL12 is moreover expected to shift the Th2 imbalance in the lymphoma lesion towards Th1 reactivity which might counteract the T cell hypo-responsiveness in Hodgkin's lymphoma. This is in line with the observation that IL12 knockout mice show enhanced Th2 responses while Th1 cell development and NK mediated lysis are deficient [20]. Decreased ability to produce IL12, as it occurs due to an IL12 polymorphism, and concomitant Th2 polarization is a necessary, although not sufficient determinant for increased susceptibility in young adult Hodgkin's lymphoma [21]. Low IL12 levels result in a reduced cellular immune response during the disease [22]; targeted IL12 is assumed to reverse the situation locally in the lymphoma lesion. IL12 is not only crucial in stimulating cytotoxic T cell and NK cell activities but also in antigen processing and presentation. Targeted IL12 is therefore of benefit to induce a strong cell based immune response with enhanced tumor cell killing [23],[24] and with high amounts of secreted IFN-γ that is required for an efficient anti-tumor response [25]–[27].

Whereas antibody-cytokine fusion proteins are explored in phase II studies for the therapy of solid cancer [28], in hematologic diseases tumor-targeted cytokines did not largely enter clinical trials. Several cytokine fusion proteins are currently explored in pre-clinical models for the immunotherapy of Hodgkin's lymphoma. Antibody targeting of IL2 to the lymphoma-associated subendothelial extracellular matrix of the neo-vasculature accumulated IL2 in the lymphoma lesions after systemic application resulting in improved anti-tumor activity compared with un-conjugated IL2 [29]. Together with the CD20 targeting antibody rituximab, the antibody-targeted IL2 improved infiltration and activation of immune effector cells in lymphoma lesions resulting in the eradication of those lesions that were not cured with rituximab alone. Targeted IL2 promotes the recruitment of NK cells and macrophages into lymphoma lesions sustaining the anti-lymphoma activity of the anti-CD20 antibody [29]. Our data indicate that the dual cytokine fusion protein, in comparison to the IL2 fusion protein, displays the synergistic action of IL2 and IL12 which might improve activation of recruited innate immune cells inside the targeted lesion.

The emerging knowledge on newly discovered IL12 family members, IL23 and IL27, may provide us with a strategy for regulating Th1 immune cell reactivation without induction of chronic inflammation. Both IL23 and IL27 have shown efficacy in treating mouse tumors [30],[31]. Since IL23 is involved in the induction of inflammatory diseases which may contribute to tumor progression, however, a combination of those cytokines with other pro-inflammatory cytokines like IL2 needs to be carefully designed.

In conclusion we have demonstrated that IL12-IL2 dual cytokine targeting to lymphoma cells provides a highly effective approach to improve activation of innate immune cells in a locally restricted fashion. Targeting of cytokine combinations may lead to a more durable and effective anti-tumor response than obtained with a single tumor-targeted cytokine alone. We believe that our findings justify the clinical evaluation of this unique combination of targeted cytokines for the therapy of CD30^+^ malignancies and may stimulate the development of a new generation of targeted fusion proteins with multiple, synergistically acting cytokines.

## Materials And Methods

### Antibodies And Reagents

The anti-mouse IL12 mAb C15.6, the biotinylated anti-mouse IL12 mAb C17.8 that detect different epitopes on murine IL-12, the anti-human IFN-γ antibody mAb NIB42, the biotinylated anti-human IFN-γ mAb 4S.B3, the anti-human IL2 mAb 5344.111, the biotin-labelled anti-human IL2 mAb B33-2 and non-conjugated as well as fluorochrome-conjugated isotype control mAbs, were purchased from BD Biosciences, Heidelberg, Germany. Biotin-labelled and unlabelled goat anti-human antibodies were purchased from Southern Biotechnology (Southern Biotechnology, Birmingham, Al, USA). The hybridoma cell line HRS3 produces the anti-CD30 mAb HRS3 [32]. Generation of the 9G10 hybridoma cell line producing the HRS3-specific anti-idiotypic mAb and of the C10 hybridoma cell line were described earlier [12],[33]. The CD30-specific single chain antibody HRS3-scFv was derived from the anti-CD30 mAb HRS3 as described [34]. The recombinant CD30-human Fc fusion protein was previously described [35] and purified from supernatants of transfected CHO cells by affinity chromatography on anti-human IgG agarose (Sigma, Deisenhofen, Germany). Monoclonal antibodies were purified from hybridoma supernatants by affinity chromatography on anti-mouse IgG agarose (Sigma). Recombinant murine IL12 and IL2 were purchased from BD Biosciences, Heidelberg, Germany.

### Cell Lines

L540cy is a CD30^+^ human Hodgkin's lymphoma cell line [36]. The murine cell line MC38 is a methylcholanthrene-induced adenocarcinoma [37]. CD30 transfectants of MC38 were generated by DNA transfection of the pCDNA3.1/V5-His TOPO vector (Invitrogen, Karlsruhe, Germany) containing the cDNA of full length human CD30. 293T (ATCC CRL-11268) is a transformed human embryonic kidney cell line and was obtained from ATCC Rockville, MD, USA.

### Generation And Expression Of Anti-Cd30 Fusion Proteins

The HRS3-scFv-Fc fusion protein and the targeted cytokines HRS3-scFv-Fc-IL2, HRS3-scFv-Fc-IL12, HRS3-scFv-IL12 and HRS3-scFv-IL2 were previously described [17],[23],[38]. To generate the HRS3-scFv-IL12-Fc and the targeted HRS3-scFv-IL12-Fc-IL2 dual cytokine fusion protein the cDNA for murine hinge-IL12 was amplified and flanked with BamHI and BglII restriction sites by the following oligonucleotides: mIL12-hinge-5′[GAAGGATCCCGCCGAGCCCAAATCTCCTGACAAAACTCATACATGCCCACCAATGTGGGAGCTGGAGAAAGACGTT]3′ and mIL12-BglII-AS-5′[GAGCTGAAGATCTCCGGCGGAGCTCAGATAGCCCATCAC]3′ (restriction sites are underlined). The PCR product was ligated into the BamHI site of the cDNA for the HRS3-scFv-Fc and HRS3-scFv-Fc-IL2 fusion proteins, respectively, thereby constituting the cDNA for the HRS3-scFv-IL12-Fc and HRS3-scFv-Fc-IL12-Fc-IL2 fusion proteins, respectively, in the pRSV expression vector. The HRS3-scFv-Fc-IL12p40 fusion protein was generated as follows: the Fc-IL12p40 fragment was amplified from pRSV-HRS3-scFv-Fc-IL12 utilizing the oligonucleotides humIgG5-Bam 5′[CTGAAGGATCCCGCCGAGCCCAAATCTCCTGACAAAACT]3′ and mp40-AS 5′[ACTTCTCTCGAGCTAGGATCGGACCCTGCAGGGAACACA]3′, thereby flanked with BamHI and XhoI restriction sites. The PCR product was digested and ligated into the BamHI and XhoI sites of the vector pRSV-HRS3-scFv-Fc thereby replacing the Fc domain of the HRS3-scFv-Fc fusion protein and constituting the expression vector pRSV-HRS3-scFv-Fc-IL12p40. The fusion proteins were secreted by transfected 293T cells into the culture supernatants and detected by ELISA. The concentration of fusion proteins with the same human IgG constant domain were adjusted to same titers as described below.

### Detection Of Anti-Cd30 Fusion Proteins

Serial dilutions of cell culture supernatants containing anti-CD30 fusion proteins or equimolar amounts of purified anti-CD30 fusion proteins were incubated in microtiter plates coated with 1 µg/ml of either the anti-HRS3 idiotypic mAb 9G10, an isotype IgG1 control antibody or the CD30-Fc antigen. Bound proteins were detected by biotinylated anti-mouse IL12 and anti-human IL2 antibodies (each 0.5 µg/ml), respectively, or a biotinylated anti-human IgG1 antibody (0.05 µg/ml) and visualized by peroxidase-coupled streptavidin (1∶10,000) and ABTS® as substrate (Roche Diagnostics). Binding specificity and efficacy, respectively, of the anti-CD30 fusion proteins was confirmed by binding competition with the CD30-Fc antigen: Purified fusion protein (10 nM of each) were incubated in microtiter plates that were coated with the CD30 antigen (1 µg/ml). Serial dilutions of the CD30-Fc antigen were added and bound fusion proteins detected by anti-IL12 and anti-IL2 antibodies, respectively, as described above. Binding inhibition was calculated utilizing the following formula:
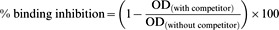
(1)To determine the titer of supernatants containing anti-CD30 fusion proteins that harbor a Fc constant domain we utilized an anti-human IgG1 antibody (0.5 µg/ml coating concentration) for capture and a biotinylated anti-human IgG1 antibody (0.05 µg/ml) for detection. The ELISA was visualized as described above. For quantitation of the supernatants a human IgG1 standard was utilized.

### Sds-Page, Bn-Page And Western Blot Analysis

Supernatants containing anti-CD30 fusion proteins were separated either by SDS-PAGE in 8% (w/v) polyacrylamide gels under reducing and non-reducing conditions or by blue native polyacrylamide gel electrophoresis (BN-PAGE) [39] and subsequently blotted onto a PVDF membrane (Invitrogen). The membrane was probed with the biotinylated anti-mouse IL12 mAb C17.8 (BD Bioscience) (0.1 µg/ml) and horseradish peroxidase conjugated streptavidin (POD) (1∶10,000) (Roche). Alternatively, the mouse anti-IL2 mAb B-G5 (0.1 µg/ml) (Serotech, Oxford, UK) and a POD labelled rabbit anti-mouse IgG (1∶10,000) (Dako, Hamburg, Germany) were used. Bands were visualized by chemiluminescence utilizing the “ECL Western blotting detection system” (Amersham Biosciences, Freiburg, Germany).

### Purification Of The Anti-Cd30 Antibody Cytokine Fusion Proteins

Anti-CD30 fusion proteins were purified from cell culture supernatants by affinity chromatography utilizing the sepharose-coupled anti-HRS3 idiotypic mAb 9G10. Briefly, mAb 9G10 (1 mg/ml in PBS, pH 7.8) was incubated for 1 h at 37°C with N-hydroxy-succinimide-ester-(NHS)-activated sepharose (Amersham Biosciences, Freiburg, Germany) according to the manufacturer's recommendation. Cell culture supernatants of transfected 293T cells were incubated with sepharose coupled 9G10 mAb and bound material was eluted with 0.1 M glycine, pH 3.0. Eluted protein was dialysed against PBS, pH 7.4, and the amount of protein was determined utilizing the Advanced Protein Assay Reagent™ (Cytoskeleton, Denver, CO, USA) according to the manufacturer's recommendations.

### Isolation Of T Cells And Nk Cells

Peripheral blood lymphocytes were isolated by density centrifugation. CD16^+^CD3^−^ NK cells and CD16^−^CD3^+^ T cells were negatively enriched by magnetic activated cell sorting (MACS) utilizing T cell and NK cell isolation kits (Miltenyi Biotech, Bergisch Gladbach, Germany). T cells were activated by anti-CD3 and IL-2 stimulation [23]. For some experiments, T cells were rested for 48 hrs in medium without stimulating agents.

### Flow Cytometry

The number of NK and T cells was determined by flow cytometry utilizing FITC-conjugated anti-CD16 and PE-conjugated anti-CD3 mAbs (Dako). Binding of the anti-CD30 fusion proteins to CD30^+^ L540cy cells was detected by a PE-conjugated F(ab)_2_ anti-human IgG1 antibody (Southern Biotechnology) and analyzed by flow cytometry using “Cell Quest” software (Becton Dickinson, Mountain View, CA).

### Lymphocyte Stimulation

Pre-activated T cells or resting NK cells (5×10^4^ cells/ml), respectively, were cultivated for 72 hrs in 24-well flat bottom plates in the presence of serial dilutions of equimolar amounts of the fusion proteins. For control, cells were cultivated in presence of human IL2 or murine IL12 (both Endogen, Woburn MA, USA). The number of living cells was determined by trypan blue staining. Culture supernatants were analysed for IFN-γ secretion by ELISA [23]. To test whether bound fusion proteins activate effector lymphocytes CD30^+^ and CD30^−^ MC38 cells were incubated with equimolar amounts of the fusion proteins, washed extensively to remove unbound protein and added (2.5×10^4^/well) to pre-activated T cells or resting NK cells (each 5×10^4^/well). Cells were co-cultivated for 48 hrs and IFN-γ secreted into the supernatants was determined. As control, MC38 cells were not washed before adding effector cells and fusion proteins remained present during co-incubation.

### Nk Cell Mediated Target Cell Lysis

Cellular cytotoxicity was monitored utilizing a XTT based colorimetric assay [40]. Briefly, resting NK cells (5×10^4^ cells/well) were co-cultivated in 96-well round bottom plates for 48 hrs together with CD30^+^ and CD30^−^ MC38 target cells (5×10^4^ cells/well) which were pre-incubated with anti-CD30 fusion proteins. To monitor cell viability, cells were incubated with XTT reagent (2,3-bis(2-methoxy-4-nitro-5sulphonyl)-5[(phenyl-amino)carbonyl]-2H-tetrazolium hydroxide; 1 mg/ml; “Cell Proliferation Kit II”, Roche Diagnostics) for 30–90 min at 37°C. Reduction of XTT to formazan by viable tumor cells was monitored colorimetrically at an absorbance wavelength of 450 nm and a reference wavelength of 650 nm. Maximal reduction of XTT was determined as the mean of 6 wells containing tumor cells only, the background as the mean of 6 wells containing RPMI 1640 medium, 10% (v/v) FCS. The non-specific formation of formazan due to the presence of effector cells was determined from triplicate wells containing effector cells in the same number as in the corresponding experimental wells. Viability of tumor cells was calculated as follows: viability [%] = [OD_(exp. wells – corresponding number of effector cells)_/OD_(tumor cells without effectors - medium)_]×100. Data were analyzed by Student's T-test.

### Radio-Iodination Of Anti-Cd30-Il12 Fusion Proteins

^131^I was purchased from Nordion (Belgium) through Bristol-Myers Squibb (BMS, Germany). Fusion proteins were labelled with ^131^I using the chloramine-T (Merck, Darmstadt, Germany) method to a specific activity of ∼500 MBq/mg. 48 MBq ^131^I was added to a solution of 20 µg fusion protein in 300 µl phosphate-buffered saline (PBS; pH 7.4) followed by 40 µl chloramine-T solution (100 µg/ml PBS). After 3 min the labelling solution was transferred to a PD-10 column (Amersham), previously rinsed with 30 ml PBS and then eluted with PBS. The eluant was collected in fractions of 1 ml. The bulk of radio-labelled protein eluted in fraction 4 which contained 22% of the starting activity. The radiochemical purity of this fraction was rechecked by thin layer chromatography using ITLC-SG strips (Gelman). Before injection into mice, radio-labelled anti-CD30-IL-12 fusion proteins were filtered using a 0.22-µm membrane filter and diluted in 20 ml PBS with 0.5% human serum albumin. ^131^I-labelled fusion proteins were also assayed for immunoreactivity. For this purpose 100 µl anti-HRS-3-idiotypic antibody 9G10 or a mouse IgG1 myeloma protein for control were coated onto ELISA plates at a concentration of 3 µg/ml. Radio-labelled anti-CD30-IL-12 fusion proteins were incubated in triplicates for 1 hour at 22°C binding of ^131^I-labelled anti-CD30-IL-12 fusion proteins was determined.

### Biodistribution Experiments

Six weeks old CB-17 SCID mice received s. c. injections with 2.5×10^7^ CD30^+^ L540cy tumor cells in the right flank. After growth of visible tumors with a diameter of about 5 mm, mice were grouped and received i.v. injections of ^131^I labelled anti-CD30-IL-12 fusion proteins. Mice were sacrificed after 24 h or 72 h after administration of the fusion protein. The organs were recovered, weighed and the radioactivity (cpm) was counted. Targeting results of representative organs are expressed as percent of injected dose/gram of tissue (%ID/g). We estimated biological half-life in blood by linear regression from % ID/g values using equations for an i. v. Bolus in an one-compartment pharmacokinetic model: *ln*(c_t_) = *ln*(c_0_)-k*t and t_1/2_ = *ln*(2)/k. This equation is also valid for two-compartment models when drug distribution between compartments has reached equilibrium. Tissue retention after 72 h was calculated as follows:To determine the biological half-life of the dual IL12-IL2-antibody fusion protein we injected three doses of 500 pmol of the HRS3-scFv-IL12-Fc-IL2 fusion protein every second day intravenously into mice. Serum samples were taken and the amount of the fusion protein was determined by ELISA as described above. We estimated biological half-life of the dual cytokine fusion protein by approximation of calculated to detected serum concentrations using the equations for an i. v. bolus in an one-compartment model as described above.

### Suppression Of Tumor Growth

Balb/c mice (Charles River, Sulzfeld, Germany) (5–10 animals/group) were subcutaneously transplanted with syngenic 9G10 or C10 hybridoma cells (2.5×10^6^ cells/animal). Seven days after tumor transplantation mice with established tumors received three i.v. injections every second day of either 50 µg/animal of the anti-CD30 IL12-IL2 dual cytokine fusion protein or saline for control. Tumor growth was recorded every 2–3 days and tumor volume determined. Statistical significance between groups was determined by the Student's T test.
